# The Influence of the Furan and Maleimide Stoichiometry on the Thermoreversible Diels–Alder Network Polymerization

**DOI:** 10.3390/polym13152522

**Published:** 2021-07-30

**Authors:** Ali Safaei, Seppe Terryn, Bram Vanderborght, Guy Van Assche, Joost Brancart

**Affiliations:** 1 Physical Chemistry and Polymer Science, Department of Materials and Chemistry, Vrije Universiteit Brussel, Pleinlaan 2, B-1050 Brussels, Belgium; ali.safaei@vub.be (A.S.); seppe.terryn@vub.be (S.T.); guy.van.assche@vub.be (G.V.A.); 2 Brubotics, Department of Mechanical Engineering, Vrije Universiteit Brussel and Imec, Pleinlaan 2, B-1050 Brussels, Belgium; bram.vanderborght@vub.be

**Keywords:** Diels–Alder, reversible polymer networks, dynamic covalent bond, self-healing, reaction kinetic simulations

## Abstract

In recent work, the thermoreversible Diels–Alder reaction between furan and maleimide functional groups has been studied extensively in the context of self-healing elastomers and thermosets. To elaborate the influence of the stoichiometric ratio between the maleimide and furan reactive groups on the thermomechanical properties and viscoelastic behavior of formed reversible covalent polymer networks, a series of Diels–Alder-based networks with different stoichiometric ratios was synthesized. Differential scanning calorimetry (DSC), dynamic mechanical analysis (DMA) and dynamic rheology measurements were performed on the reversible polymer networks, to relate the reversible network structure to the material properties and reactivity. Such knowledge allows the design and optimization of the thermomechanical behavior of the reversible networks for intended applications. Lowering the maleimide-to-furan ratio creates a deficit of maleimide functional groups, resulting in a decrease in the crosslink density of the system, and a consequent decrease in the glass transition temperature, Young’s modulus, and gel transition temperature. The excess of unreacted furan in the system results in faster reaction and healing kinetics and a shift of the reaction equilibrium.

## 1. Introduction

In the context of step-growth polymerization of linear polymers, the stoichiometric ratio between reactive groups is well-known as a direct approach to control the number average molar mass, while more generally speaking, the combination of monofunctional and multifunctional monomers and the ratios thereof determine the polymer architecture formed [1]. For chain-growth polymerizations, the ratios between initiator, monomer and/or chain transfer agent determine the molecular weight [2]. In both linear cases and in the case of network-forming polymerizations, the thermophysical and thermomechanical properties depend on the formed polymer architectures. The Diels–Alder reaction, described by Otto Diels and Kurt Alder [3] is one of the most well-known and widely used thermoreversible equilibrium reaction to construct polymer networks with intrinsic self-healing ability [4,5,6]. The most studied Diels–Alder (DA) reaction is the cycloaddition of furan, a conjugated electron rich diene, and maleimide, an electron poor dienophile, forming a DA cycloadduct. The reverse process, called the retro Diels–Alder (rDA) reaction, converts the cycloadduct into the starting diene and dienophile [7]. Their sufficiently fast reaction kinetics and high conversion at room temperature [8] make them suitable candidates for thermoresponsive materials, such as, thermoremendable and self-healing polymer networks, which can be processed and healed at temperatures between 80 and 140 °C due to the thermoreversible crosslinking. Reversible polymer networks (RPN) constructed by DA reactions consist of covalently reversible chemical crosslinks that can be broken upon an external stimulus, mainly heat or radiation energy, and consequently damage can be healed via a heat-cool cycle below the degelation transition, while reshaping and reprocessing is feasible above degelation transition. These reversible networks have been proven to be valuable in robotics applications [9,10,11,12,13] by increasing the life-time of components through healing of macroscopic damages and as protective coatings [14,15,16]. Multiple synthesis design parameters allow tuning the mechanical and processing properties of Diels–Alder networks.

To tune the mechanical properties of the dynamic covalent polymer networks for an intended application, different strategies can be used. For elastomers, it is well established how the molecular weight and flexibility of the chain segments between the crosslinking nodes affect the crosslink density and resulting mechanical properties of the polymer networks. The effect of the concentration of the furan and maleimide functional groups on the Diels–Alder reaction kinetics, thermodynamics and viscoelastic properties and thermomechanical behavior have been well studied for stoichiometric ratios by the authors and peers [17]. Examples in literature exist where the stoichiometry between maleimide and furan functional groups was varied, to study the reaction kinetics [8], to alter the mechanical properties for multi-material self-healing actuators [10,11], and to improve the healing performance to achieve self-healing at ambient conditions [12]. In general, increasing the concentration of the furan groups to an excess is expected to speed up the self-healing reactions and to drive the Diels–Alder equilibrium more to the bound state, however, the resulting dangling chain segments of the unreacted furans could negatively affect the mechanical behavior. Therefore, we investigated the influence of the stoichiometric ratio on the viscoelastic properties and thermomechanical behavior, including the glass transition and gel transition temperatures, Young’s modulus and stress–strain behavior, as well as on the reaction kinetics and equilibrium of synthesized reversible covalent polymer networks. It is illustrated that starting from only two specific monomers, a bismaleimide and a furan functionalized Jeffamines, a wide variety of polymer networks can be synthesized with mechanical properties ranging from very stiff to hyper elastic, by altering the stoichiometric ratio. Consequently, using only two monomers, the material properties can be fitted to meet requirements imposed by the manufacturing technique or the application.

## 2. Materials and Methods

### 2.1. Materials

Furfuryl glycidyl ether (FGE, 96%) was obtained from Sage Chemicals (Hangzhou, China). Jeffamine D400 with an average molecular weight of 432 g mol^−1^ was supplied by Huntsman (Everberg, Belgium). 1,1′-(methylenedi-4, 1-phenylene) bismaleimide (DPBM, 95%) was obtained from Sigma Aldrich. Hydroquinone (1, 4-benzenediol, 99%) was used as a radical inhibitor and was supplied by Sigma Aldrich (Overijse, Belgium). All chemicals were used as received.

### 2.2. Synthesis

**Figure 1:**
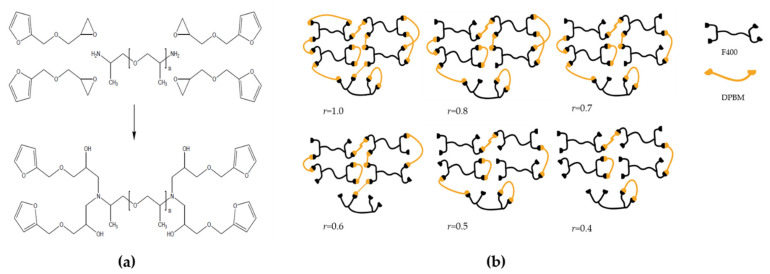
Two steps of synthesizing reversible polymer networks: (**a**) preparation of a four-functional furan compound through an epoxy-amine reaction between a Jeffamine D series amine (D400) and furfuryl glycidyl ether (FGE); (**b**) schematic of the reaction products of the furan functionalized Jeffamine (F400) with bismaleimide (DPBM) at full maleimide conversion for different maleimide to furan ratios (*r*).

The synthesis of the reversible covalent polymer networks is performed in two steps [18]. First, furfuryl glycidyl ether (FGE) is reacted with Jeffamine D400 through an irreversible epoxy-amine reaction. This reaction should be carried out under stoichiometric conditions between amine hydrogens and epoxy functional groups. The container is placed in an oil bath at 60 °C and magnetically stirred for 5 days. Then the temperature is raised to 90 °C for 2 days to complete the epoxy-amine reaction. In a second step, the furan-functionalized Jeffamine is reacted with the bismaleimide (DPBM) crosslinker to form the Diels–Alder adducts as reversible crosslinks for the reversible covalent network. To facilitate the mixing of the reagents, chloroform (CHCl_3_) is used as a solvent. To prevent homopolymerization of the maleimide at high temperature, hydroquinone (5 wt % of DPBM) is added to the mixture as a radical inhibitor. To ensure excellent mixing and complete dissolution of the solids, the mixture is stirred for 24 h. The network is formed through solvent casting in which the chloroform is extracted via vacuum oven at room temperature for another 24 h. During evaporation, the concentration of maleimide and furan is increased, pushing the equilibrium towards the formation of DA crosslinks and the formation of the network (Figure 1b).

### 2.3. Instruments

#### 2.3.1. Differential Scanning Calorimetry

To determine the glass transition temperature (*T*_g_) of the synthesized Diels–Alder networks a TA Instruments Q2000 DSC equipped with a Refrigerated Cooling System (RCS) was used. Nitrogen was used as a purge gas. Heat-cool cycles were performed between −80 °C and 120 °C at a rate of 10 K min^−1^. The materials stayed isothermal for 5 min at −80 °C and 1 min at 120 °C. Samples having a mass of 5–8 mg were placed in aluminium T_zero_ pans (TA Instruments), subsequently sealed with a hermetic lid (TA Instruments).

#### 2.3.2. Dynamic Mechanical Analysis

Dynamic mechanical analysis (DMA) was carried out using a TA Instruments DMA Q800 equipped with a liquid nitrogen gas cooling accessory. Stress–strain tensile tests were performed at room temperature in controlled strain mode using a film tension clamp (TA Instruments). Rectangular specimens (about 14 mm by 4.55 mm by 0.40 mm) cut from the cast sheets were clamped with a length of 7.5 mm and were subjected to a tensile strain increasing at a rate of 1% min^−1^ with 0.01 N preload force and 0.01% initial strain (Strain Rate mode). The tensile properties were determined from the average values of at least five replicates for each material. The Young’s modulus was determined in the initial linear region of the stress–strain curve (0–0.5% strain). Small amplitude oscillatory measurements were performed to study the viscoelastic properties (storage modulus *E*′, loss modulus *E″*, and tangent of the loss angle δ) at a frequency of 1 Hz and 0.1% strain in heat-cool cycles at a rate of 2 K min^−1^ between −30 °C and 110 °C in Multi-frequency-Strain mode, using a Track Force setting of 115%.

#### 2.3.3. Dynamic Rheometry

Dynamic rheometry is performed with a TA Instruments Discovery Hybrid Rheometer (DHR2) to determine the (de)gelation temperature. The experiment is performed using a 10 mm aluminium parallel plates geometry in a temperature range from 80 °C to 140 °C and heating rate of 1 K min^−1^ with 5 min soaking time at 80 °C. The sample (10 mm diameter, 1 mm thickness) was subjected to an oscillatory strain with an amplitude of 5% at different frequencies: 0.312, 0.562, 1.0, 1.778, and 3.125 Hz.

## 3. Results

Varying the stoichiometric ratio of the reactive functional groups in a crosslinking polymerization results in changes in the thermophysical and thermomechanical properties of the formed network, as reported in literature for irreversible network polymerizations, such as epoxy-amine reactions [19,20,21] and polyester formation [22]. Moreover, changing the (ratio between the) concentrations of the functional groups results in important changes in the kinetics of the polymerization reactions. In case of transesterification-based vitrimers, it was reported how changing the stoichiometric ratio between the formed ester bonds and excess hydroxyl groups greatly influences the exchange kinetics of the transesterification reaction [23,24,25]. This work details the influence of the stoichiometric ratio between the furan and maleimide functional groups on the reaction kinetics and equilibrium, the thermomechanical properties, and the self-healing behavior of the formed thermoreversible covalent polymer networks. To do so, six DA networks were synthesized that differ in stoichiometric ratio (*r* = [*M*]_0_/[*F*]_0_). The initial maleimide [*M*]_0_ and furan [*F*]_0_ concentrations are presented in Table 1.

### 3.1. Effect of Stoichiometric Ratio on Crosslink Density

The stoichiometric ratio affects the crosslink density of the produced networks. This is illustrated by the Diels–Alder adduct [*DA*]*_eq_*, maleimide [*M*]*_eq_* and furan [*F*]*_eq_* concentration at equilibrium at 25 °C presented in Table 1. These equilibrium concentrations were calculated based on the rate constants of the forward and retro *DA* reactions. Actually, during the *DA* reaction, two stereoisomeric *DA* adducts (*endo* and *exo*) are generated. Therefore, the kinetics of the equilibrium reaction are described by four rate constants (*k_DA,endo_*, *k_rDA,endo_*, *k_DA,exo_*, *k_rDA,exo_*):

$$DA_{endo}\;\begin{array}{c} k_{DA,endo}\\ ⇋\\ k_{rDA,endo} \end{array}\;F+M\;\begin{array}{c} k_{DA,exo}\\ ⇌\\ k_{rDA,exo} \end{array}DA_{exo}$$

The rate constants for the formation of *endo* and *exo* adduct and the reverse Diels–Alder reactions can be calculated using the Arrhenius law (Equation (1)).

(1) $$k_{DA,i}=A_{DA,i}e^{\frac{-E_{DA,i}}{RT}}\;\;\;\;\;\;\;\;\;\;\;\;\;\;\;\;\;\;k_{rDA,i}=A_{rDA,i}e^{\frac{-E_{rDA,i}}{RT}}$$

In these equations, “*i*” stands for *endo* or *exo*, *A_DA,i_* and *A_rDA,i_* are pre-exponential factors or frequency factors, and *E_DA,i_* and *E_rDA,i_* are activation energies. The values for these kinetic parameters can be found in Table A1. *R* is the universal gas constant and *T* the absolute temperature. The rate of change of the concentration of reactants and adducts is described by the differential equations given in Equations (2) and (3):

(2) $$\frac{d\left[M\right]}{dt}=\frac{d\left[F\right]}{dt}=-\frac{d\left[DA\right]}{dt}=\underset{i=exo,endo}{\sum }\left\{\;k_{rDA,i}\left[DA,i\right]-k_{DA,i}\left[M\right]\left[F\right]\;\right\}$$

(3) $$\frac{d\left[DA,i\right]}{dt}=\;k_{DA,i}\left[M\right]\left[F\right]-\;k_{rDA,i}\left[DA_{i}\right]\;\;\;$$

The overall reaction conversion *x* is defined as the extent of the reaction, based on the initial concentration of maleimide, the minority component:

(4) $$x=\frac{\left[DA\right]}{{\left[M\right]}_{0}}$$

[DA] represents the sum of the concentrations of the two adducts. The apparent equilibrium constant *K_C_*_,*DA*_, defined in (Equation (5)), is not a real equilibrium constant, but the sum of the equilibrium constants for the formation of the *endo* and *exo* adducts, based on the rate constants *k_DA,i_* and *k_rDA,i_*.

(5) $$\begin{array}{c} K_{C,DA}=K_{C,\;endo}+K_{C,\;exo}=\;\;\frac{k_{DA,endo}\;C_{0}}{k_{rDA,endo}}+\frac{k_{DA,exo}\;C_{0}}{k_{rDA,exo}}\\ =\frac{{\left[DA_{endo}\right]}_{eq}C_{0}+{\left[DA_{exo}\right]}_{eq}C_{0}}{{\left[F\right]}_{eq}{\left[M\right]}_{eq}}=\frac{x_{eq}C_{0}}{r{\left(1-x_{eq}\right)}^{2}{\left[F\right]}_{0}} \end{array}$$

Solving this equation towards the equilibrium conversion *x_eq_*, gives Equation (6). The mathematical operations involved are given in Table A1. Equation (6) is an extension of formulas reported earlier by the authors [16], taking into account the stoichiometry of the reactive groups.

(6) $$x_{eq}=\;\frac{K_{C,DA}\left(1+\frac{1}{r}\right){\left[M\right]}_{0}+1-\sqrt{{\left(K_{C,DA}\left(1+\frac{1}{r}\right){\left[M\right]}_{0}+1\right)}^{2}-\frac{4K_{C,DA}{}^{2}{\left[M\right]}_{0}{}^{2}}{r}}}{2K_{C,DA}{\left[M\right]}_{0}\left(\frac{1}{C_{0}}\right)}$$

This equation allows one to calculate the maleimide, furan and DA adduct concentrations at equilibrium for any temperature using Equation (7):

(7) $${\left[DA\right]}_{eq}=x_{eq}{\left[M\right]}_{0}\;\;\;\;\;\;{\left[M\right]}_{eq}=(1-x_{eq}){\left[M\right]}_{0}\;\;\;\;\;\;{\left[F\right]}_{eq}=(1-rx_{eq}){\left[F\right]}_{0}$$

Table 1 shows how the starting concentrations of the maleimide and furan functional groups change with lowering the stoichiometric ratio (*r*), and how this affects the equilibrium cycloadduct concentration that can still be achieved. The equilibrium concentrations of maleimide, furan and DA adducts at a reference temperature of 25 °C were calculated using Equations (6) and (7). It is clear that decreasing the stoichiometric ratio, decreases the crosslink density of the produced networks. At *r* equal to one, the number of furan and maleimides groups in the monomer system is equal and the highest concentration of DA cycloadducts can be formed. Hence, the highest crosslink density is obtained, as depicted schematically in Figure 1. If a lower stoichiometric ratio is selected, the maleimide groups are in deficit. The amount of adducts that can still be formed depends on the limiting concentration of the maleimide groups. Consequently, networks with a low stoichiometric ratio have a lower crosslink density and a larger amount of unreacted furan, as schematically illustrated in Figure 1. The series of DA networks presented in Table 1 was characterized via differential scanning calorimetry (DSC), dynamic mechanical analysis (DMA), and dynamic rheology measurements, to illustrate the effect of the *r* ratios on the material properties, as described in the following sections.

**Figure 2:**
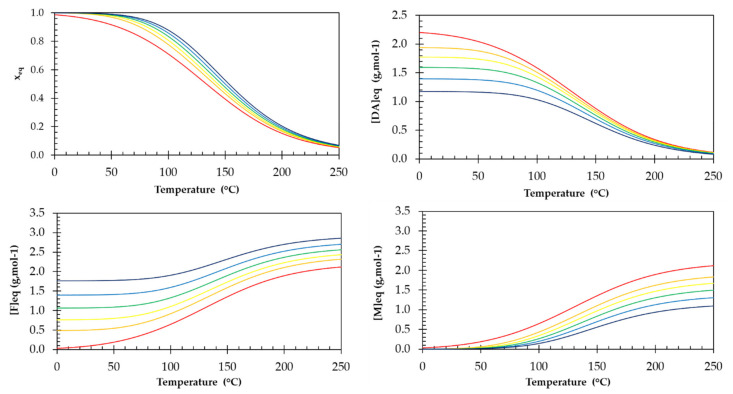
The simulated equilibrium conversion *x_eq_* and the equilibrium concentrations [*DA*]*_eq_*, [*M*]*_eq_* and [*F*]*_eq_* as a function of the temperature for the series of six DA networks that differ in stoichiometric ratio: *r* = 1 (red), *r* = 0.8 (orange), *r* = 0.7 (yellow), *r* = 0.6 (green), *r* = 0.5 (light blue), and *r* = 0.4 (dark blue).

In contrast to traditional thermosetting elastomers, the crosslink density of these reversible networks decreases with increasing temperature. To illustrate this effect, for each of the six materials the equilibrium conversion *x_eq_* and the concentrations [*DA*]*_eq_*, [*M*]*_eq_* and [*F*]*_eq_* were calculated via Equations (6) and (7) for the 0 to 250 °C temperature window (Figure 2). As the temperature increases, the reaction rates of both forward and retro DA reactions increase, however, due to their higher activation energies the reverse reactions accelerate more, shifting the equilibrium towards the breaking of the reversible bonds. This results in a decrease in equilibrium conversion and crosslink density with increasing temperature (Figure 2). While the reversible polymer network becomes more dynamic. On the other hand, at low temperatures the reaction equilibrium is shifted towards the formation of the cycloadduct, resulting in high values for the equilibrium conversion and crosslink density. At ambient temperatures the DA reaction is predominant, and the conversion is near unity.

For decreasing *r* values, the crosslink density in the network decreases and this effect is more pronounced at lower temperatures. At ambient temperatures and below, stoichiometric networks (*r* = 1) have nearly no unreacted maleimide and furan groups, as illustrated by a high equilibrium conversion *x_eq_*. For lower *r* values, the excess of furan is higher, pushing the equilibrium towards the formation of DA adducts, which results in a higher equilibrium conversion for a given temperature (Figure 2 and *x_eq_* at 25 °C and 100 °C in Table 1).

### 3.2. Effect of the Stoichiometric Ratio on the Glass Transition Temperature

**Figure 3:**
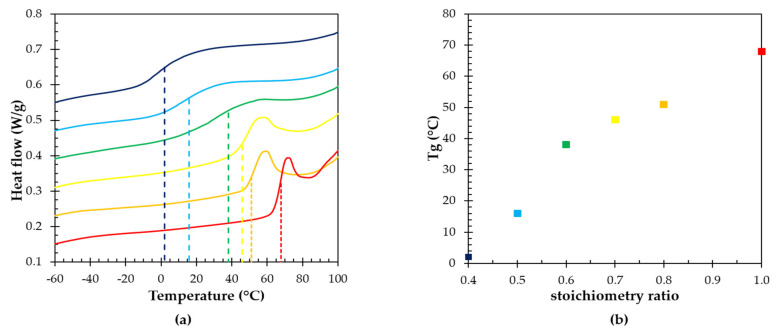
(**a**) DSC curves for thermoreversible networks; (**b**) glass transition temperature of the RPN as a function of stoichiometric ratio (*r*): *r* = 1 (red), *r* = 0.8 (orange), *r* = 0.7 (yellow), *r* = 0.6 (green), *r* = 0.5 (light blue), and *r* = 0.4 (dark blue). The glass transition is visible as an endothermic step in the heat flow signal as function of temperature. By increasing the stoichiometric ratio, the glass transition *T_g_* increases due to an increase in crosslink density.

The glass transition is a crucial property for (reversible) polymer networks, as it determines whether these amorphous materials will behave like a rubber or like a glass at a specific temperature. When heating through the glass transition, the cooperative chain segment mobility quite rapidly increases, resulting in an increase in the heat capacity, which is visible as an endothermic step in the heat flow signal in DSC (Figure 3a). The glass transition temperatures (*T*_g_) of the six RPN are shown in Figure 3b as a function of the stoichiometric ratio (*r*). At *r* equal to one, the highest concentration of Diels–Alder cycloadducts is formed, leading to the highest glass transition temperature (Table 2 and Figure 3). Lowering the stoichiometric ratio, results in lower crosslink densities and lower glass transition temperatures. Interestingly, by changing the stoichiometric ratio (initial concentration of the furan and maleimide), a wide variety of polymeric network properties can be obtained, ranging from brittle and glassy thermosets (*T_g_ > T_amb_*) to flexible, ductile elastomers (*T_g_ < T_amb_*) at ambient temperature (Figure 3).

### 3.3. Effect of the Stoichiometric Ratio on the Mechanical Properties

**Figure 4:**
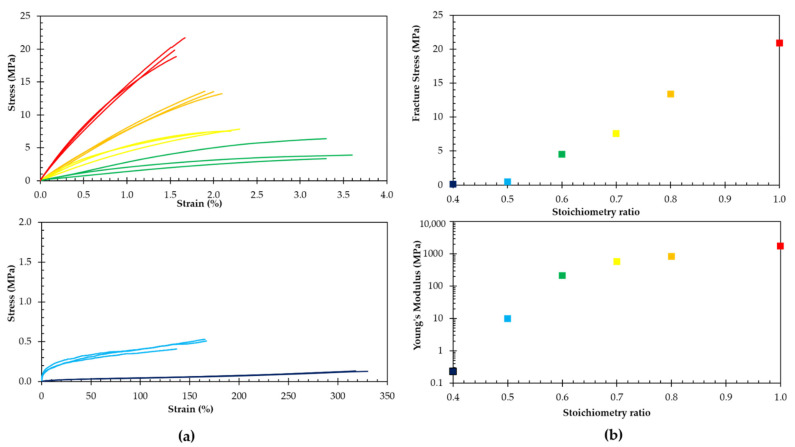
(**a**) The stress–strain curves of DPBM-F400 (*r* = 1) red, (*r* = 0.8) orange, (*r* = 0.7) yellow, (*r* = 0.6) green (**top**), and (*r* = 0.5) light blue, (*r* = 0.4) dark blue (**below**) in tension mode at a 1% min^−1^ strain rate; (**b**) fracture stress (**top**) and Young’s Modulus (**below**) as a function of stoichiometric ratio *r* of synthesized RPNs.

The mechanical properties were determined in tensile tests under ambient conditions. Figure 4 compares the stress–strain behavior of the different RPN. The Young’s moduli were calculated as a chord modulus at a strain of 0.5%, as 0–0.5% is taken as the linear region. For a lower stoichiometric ratio, the number of crosslinks is lower (Table 1) and, consequently, the flexibility increases, illustrated by a decrease in Young’s modulus, an increase in strain at break and a decrease in stress at break. The polymer with the highest stoichiometric ratio, the most densely crosslinked network, has the highest modulus of 1.7 GPa. This polymer network also exhibits a very low strain at break of around 1.6% and a stress at break of around 21 MPa. In contrast, a much less crosslinked material, DPBM-F400 (*r* = 0.5), is hyper flexible and exhibits a low Young’s modulus of 9.8 MPa and high fracture strain of 156%. The *r* = 0.4 network, was even too flexible to be tested in the tensile test setup of the DMA. This illustrates nicely that using only two specific monomers, bismaleimide DPBM and furan-functionalized Jeffamine F400, and by varying the stoichiometric ratio, a wide variety of networks can be synthesized, with a mechanical behavior at ambient condition varying from hard thermosets (*r* = 0.8–1.0) to hyper elastic elastomers (*r* = 0.4–0.5). This allows to optimize the mechanical properties of the network to fit specific demands imposed by desired applications.

### 3.4. Effect of the Stoichiometric Ratio on the Thermomechanical Behavior

#### 3.4.1. Viscoelastic Properties

**Figure 5:**
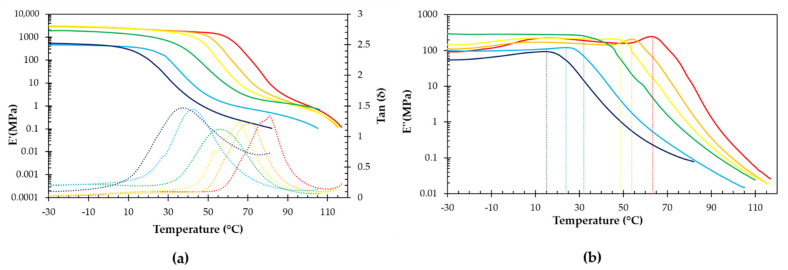
(**a**) Storage modulus *E′* (solid lines) and Tan(*δ*) (dotted lines); (**b**) Loss modulus *E″* of DPBM-F400 with different stoichiometric ratio: *r* = 1 (red), *r* = 0.8 (orange), *r* = 0.7 (yellow), *r* = 0.6 (green), *r* = 0.5 (light blue), and *r* = 0.4 (dark blue), measured at 1 Hz and 0.1% strain.

To investigate the influence of temperature on the viscoelastic properties of the six networks, DMA was used. In Figure 5, the storage modulus (*E′*), loss modulus (*E″*) and Tan(*δ*) are compared as a function of temperature. All RPN show a clear drop in mechanical properties at the glass transition, as expected when the material goes from a vitrified state to an elastomeric state. Decreasing the stoichiometric ratio leads to a decrease in crosslink density and, consequently, a decrease in storage modulus and a shift of the peak of both Tan(*δ*) and loss modulus (*E″*) to lower temperatures. According to Winter [26], the glass transition temperature (*T*_g_) is defined as the temperature at which the loss modulus reaches its local maximum. With decreasing *r* ratio, the *T*_g_ decreases, as was also observed in DSC. As the temperature continues to increase, the moduli continue to drop, first gradually in the rubbery plateau due to the continued decrease of the crosslink density, then more rapidly as the network structure is broken down more rapidly and the material starts to lose its mechanical integrity. From the viscoelastic properties at 25 °C shown in Table 3, it can be seen that the highest *r* ratio leads to a hard thermoset behavior with a storage modulus of 2023 MPa and an almost entirely energy-elastic behavior, indicated by a low loss angle of 4.7°. The lowest *r* ratio results in a flexible elastomeric behavior with a low storage modulus of 45.9 MPa and a non-negligible loss angle of 45.7°. This illustrates again the broad range of properties that can be achieved by varying this stoichiometric ratio.

#### 3.4.2. Gel Transition Temperature

**Figure 6:**
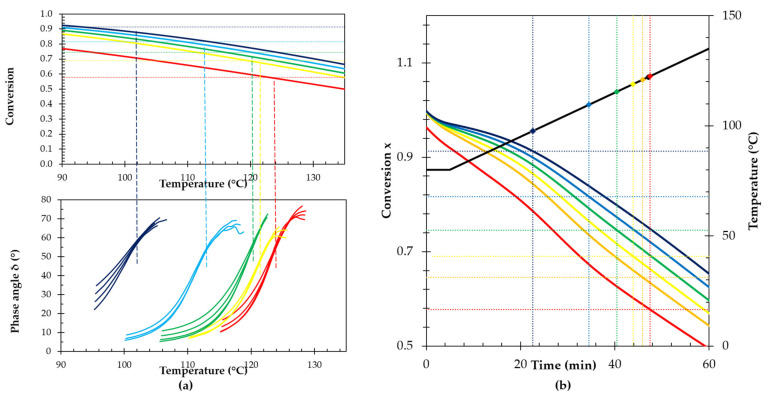
(**a**) Relating equilibrium conversions and experimental gel temperatures for DPBM-F400 with different stoichiometric ratio. (**a**–**top**) DA equilibrium conversion *x_eq_* (solid line) and gel conversion (dashed line) as a function of temperature (top). (**a**–**below**) Phase angle from multi-frequency dynamic rheometry measurements at 1 K min^−1^ with the cross-over indicating degelation; (**b**) Simulation of the non-equilibrium conversion *x* during the rheology experiment of DPBM-F400 with different stoichiometric ratio *r* = 1 (red), *r* = 0.8 (orange), *r* = 0.7 (yellow), *r* = 0.6 (green), *r* = 0.5 (light blue), and *r* = 0.4 (dark blue). Horizontal dotted lines represent the gel conversions, while vertical dotted lines indicate the time at which the network (de)gels in this experiment. The gel transition temperature is presented by a diamond dot.

In case of thermally dissociative polymer networks, a gel transition temperature *T*_gel_ can be determined at which the material behavior transitions reversibly from predominantly elastic to liquid-like and vice versa [7]. For polymer networks that can be dissociated photochemically and in some cases also thermally, a gel time can be determined at a specific light irradiation intensity or at a certain temperature [27]. The reversible gel transition is important in terms of applications and even more for the processing of the reversible network materials. At the gel point, the reaction conversion *x* (Equation (4)) equals the critical gel conversion *x*_gel_, which can be calculated from the functionalities of the monomers *f_M_* and *f_F_* and the stoichiometric ratio *r* using the Flory–Stockmayer equation [1,28] (Equation (8)). For the DPBM-F400 networks studied, *f_M_* = 2 and *f_F_* = 4 are fixed and only the stoichiometric ratio *r* is varied. Above the gel conversion (at *x* > *x_gel_*), the polymeric network behaves as a solid, since the crosslink density is high enough to form a network percolating through the material. As the thermoreversible polymer network is heated, the (equilibrium) reaction conversion decreases with temperature (Figure 2). Eventually the conversion will drop below the critical gel conversion (*x* < *x_gel_*), leading to the loss of the network connectivity and resulting in viscous flow behavior. The gel temperature *T*_gel_, the temperature at which the material passes through its gel transition, is determined in dynamic rheometry experiments as the point where the loss angle δ is independent of the oscillation frequency [26]. Figure 6a shows multifrequency dynamic rheometry experiments of the RPN with different stoichiometries. The highest *T*_gel_ was observed for the stoichiometric network. Decreasing the maleimide-to-furan ratio *r* leads to (de)gelation at lower temperatures, in agreement with a higher critical gel conversion *x_gel_*, following the Flory–Stockmayer equation (Equation (8)) and a lower equilibrium conversion with temperature (Figure 2).

(8) $$x_{gel}=\frac{1}{\sqrt{r\left(f_{M}-1\right)\;\left(f_{F}-1\right)}}$$

The equilibrium conversion *x*_eq_ for the DA reaction between furan and maleimide can be calculated as a function of temperature using Equation (6), as shown in Figure 6a for the RPNs with different *r* and, hence, different [*M*]*_0_*. The intersection of the equilibrium conversion (solid curves) and the gel conversion (dashed horizontal lines), indicates the equilibrium gel transition temperature, *T*_gel,sim(eq)_, where the equilibrium conversion *x_eq_* equals *x_gel_*. The simulated equilibrium gel transition temperatures *T*_gel,sim(eq)_ are compared to the experimental *T*_gel,exp_ in Table 4. At higher stoichiometric ratios the model is able to calculate the *T*_gel,exp_ very well, while the error becomes larger as the stoichiometric ratio decreases. This can be partially explained by the fact that at lower temperature the kinetics are slower (Equation (1)) and the network is not at chemical equilibrium at these temperatures during the rheometry experiment. To check this, the evolution of the non-equilibrium conversion during the rheometry experiment was simulated using the rate equations (Equations (2) and (3)) and the temperature-time profile of the experiment (Figure 6b). The initial concentrations for this simulation were the equilibrium concentrations at 25 °C. The non-equilibrium gel transition temperatures derived from these simulations are also presented in Table 4. These simulated *T*_gel*,*sim(non-eq)_ coincide better with the experimental values, confirming that at lower temperatures the experiment, performed at a temperature ramp of 1 K min^−1^) was not performed in near equilibrium conditions. From both simulated and experimental gel transition temperatures (Figure 6 and Table 4), it can be seen that lowering the stoichiometric ratio results in a shift to higher (equilibrium) conversion. Following Le Chatelier’s principle, an excess of furan functional groups will result in a higher maleimide conversion into cycloadduct at the same temperature and pressure. However, also the critical gel conversion *x_gel_* increases with decreasing *r* (Equation (8)). Taking both shifts into account, it can be seen that the effect of the latter is larger, ultimately resulting in a decreasing *T*_gel_ with decreasing stoichiometric ratio.

**Figure 7:**
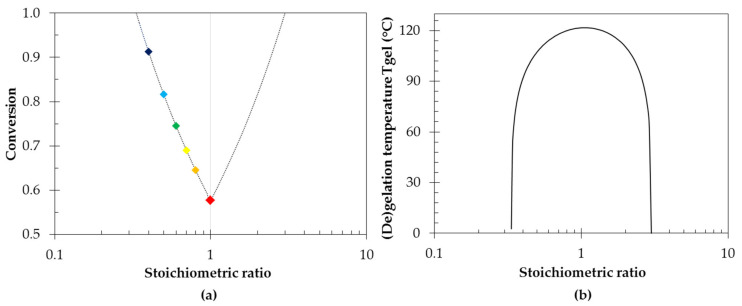
The simulated critical gel conversions (**a**) and the simulated equilibrium gel transition temperatures (**b**) for [4 + 2] networks as a function of stoichiometric ratio.

In this paper, the crosslink density is controlled by a deficit of maleimide and an excess of furan. However, similarly a deficit of furan and an excess of maleimide (*r* > 1) lead to networks with reduced crosslink density. According to the Flory–Stockmayer theory the critical gel conversion is based on the limiting component and can be calculated for different combinations of monomer functionalities as a function of their stoichiometric ratios (Equation (9)). In Figure 7a, the relation between the critical gel conversion and the stoichiometric ratio is illustrated for monomers with 4 furan and 2 maleimide functional groups. If *r* < 1 an excess amount of furan groups is present with respect to decreasing amounts of maleimide groups. In that case the conversion will be determined for the limiting maleimide groups. In the inverse case (*r* > 1), the conversion will be based on the furan groups. For a fixed functionality of monomer units, the gel conversion is the lowest for stoichiometric condition (*r* = 1). The more off-stoichiometric the reaction mixture is, the higher the conversion needed to form a network and the lower the gel transition temperature (Figure 7b).

(9) $$x_{Mgel}=\;\;\frac{1}{\sqrt{r\left(f_{M}-1\right)\left(f_{F}-1\right)}}\;\;\;\;\;\;\;x_{Fgel}=\;\;\frac{\sqrt{r}}{\sqrt{\left(f_{M}-1\right)\left(f_{F}-1\right)}}$$

### 3.5. Effect of the Stoichiometric Ratio on the Diels–Alder Kinetics

**Figure 8:**
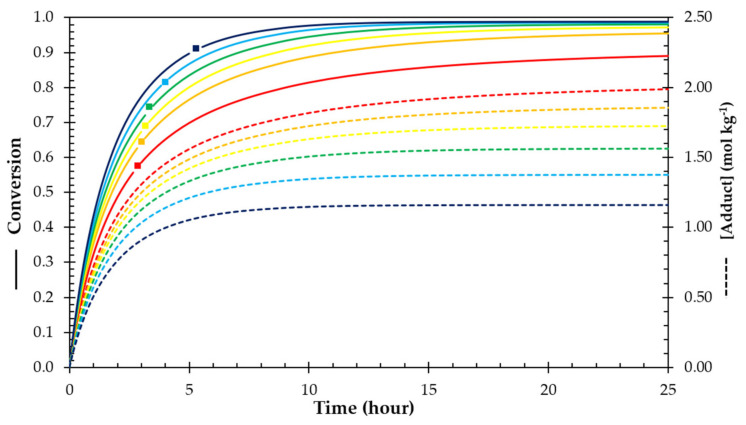
Diels–Alder conversion (solid lines), gelation time (squares), and total adduct concentration (dashed lines) for the DPBM-F400 reversible polymer network with different stoichiometry as a function of time at 25 °C: DPBM-F400 (*r* = 1) red, (*r* = 0.8) orange, (*r* = 0.7) yellow, (*r* = 0.6) green, (*r* = 0.5) light blue, and (*r* = 0.4) dark blue.

**Figure A1:**
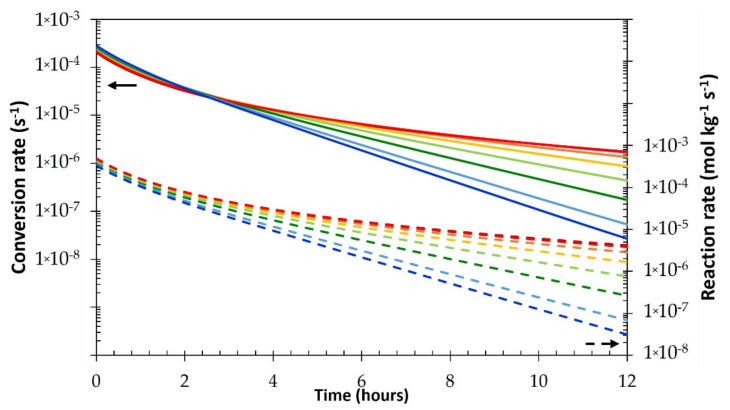
Diels–Alder conversion rate (solid lines) and reaction rate (dashed lines) for the DPBM-F400 reversible polymer network with different stoichiometry as a function of time at 25 °C: DPBM-F400(*r* = 1) red, (*r* = 0.8) orange, (*r* = 0.7) yellow, (*r* = 0.6) green, (*r* = 0.5) light blue, and (*r* = 0.4) dark blue.

Although the equilibrium conversion curves of Figure 1 are determining the equilibrium properties at different temperatures, the Diels–Alder kinetics will determine how fast the broken bonds will be reformed or how fast a material will self-heal. Figure 8 shows isothermal simulations for the Diels–Alder bond formation process at a constant temperature of 25 °C and for different stoichiometric ratios, and thus different initial concentrations. If the initial concentration of maleimide groups is decreased (lower *r* value), leading to a deficit of maleimide and an excess of furan groups in the system, the final concentration of adducts (dashed lines in Figure 8) that can be formed is limited by the maleimide, resulting in lower adduct concentrations (crosslink densities) and hence more flexible network structures, as discussed in previous sections. Conversely, the maleimide conversion (solid lines in Figure 7) is higher at all times for the RPN with the lowest stoichiometric ratio, as an excess of maleimide pushes the equilibrium towards the formation of DA adduct. The conversion rate, the slope of the solid lines in Figure 8 and also presented as the solid lines in Figure A1 in Appendix A, is higher at the start for the lowest stoichiometric ratio, and remains higher as the concentration of the furan present in excess decreases relatively less. Hence, the equilibrium conversion is reached much faster for the off-stoichiometric systems. To quantify this, the reaction times *t*_eq,95%_ to reach 95% of the equilibrium conversion at 25 °C are compared in Table 4, decreasing from 175 h for *r* = 1 to 17 h for *r* = 0.8 and decreasing further down to 6 h for *r* = 0.4. It should be noted, however, that due to the higher gel conversions of the off-stoichiometric systems, the time *t_gel_* to reach gelation increases with decreasing stoichiometric ratio (Table 4).

### 3.6. Effect of Stoichiometric Ratio on the Self-Healing Behavior

**Figure 9:**
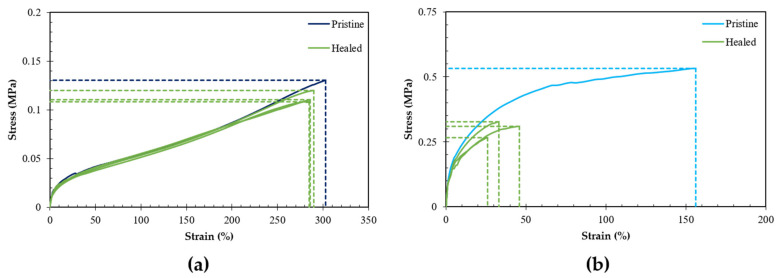
A comparison of stress–strain curve of pristine and samples were cut completely and healed for 15 h at 25 °C. (**a**) DPBM-F400(*r* = 0.4), and (**b**) DPBM-F400(*r* = 0.5).

Lowering the stoichiometric ratio showed to result in more flexible reversible polymer networks with increased Diels–Alder reaction kinetics. A change in kinetics influences the healing ability as well. To illustrate this self-healing tests were performed for networks with different stoichiometry, including DPBM-FD400 (*r* = 0.4, *r* = 0.5, *r* = 0.6 and *r* = 0.8). Samples with a gauge length of 7.5 mm, a width of 3–5 mm and a thickness of 1.5–2 mm were cut completely in half using a scalpel blade and brought back in contact seconds after damage. These tensile samples were left to heal for 15 h at 25 °C and subsequently tested in a stress–strain experiment until fracture with a strain ramp of 1%s^−1^. Comparing this stress–strain data with pristine samples, the DPBM-F400 (*r* = 0.4) shows an excellent healing behavior at 25 °C as can be seen in Figure 9a. The stress–strain behavior is completely recovered after healing for 15 h at 25 °C, resulting in a mean recovery of 91% and 95% of respectively the fracture stress and strain of the pristine material prior to damage. This excellent healing behavior is a result of the optimal combination of increased mobility due to the lower crosslink density of the polymer network (maleimide deficit) and improved reformation of the broken reversible covalent bonds due to faster kinetics as a result of a large furan excess. Further lowering of the stoichiometric ratio to values below 0.4 resulted in a material with poor mechanical properties and stability. Conversely, higher stoichiometric ratios lead to stiffer materials, with lower mobility and more importantly slower kinetics as shown in Figure 8. This is visible for the DPBM-F400 (*r* = 0.5) in Figure 9b, where under the same conditions the healing efficiency only about 68% for the stress at break and as little as around 24% for the strain at break. For the networks with higher stoichiometry (*r* = 0.6 and *r* = 0.8), the cut parts could not be healed at all and did not adhere after 15 h at 15 °C. This clearly demonstrates the limits to achieve successful self-healing under ambient conditions.

**Figure 10:**
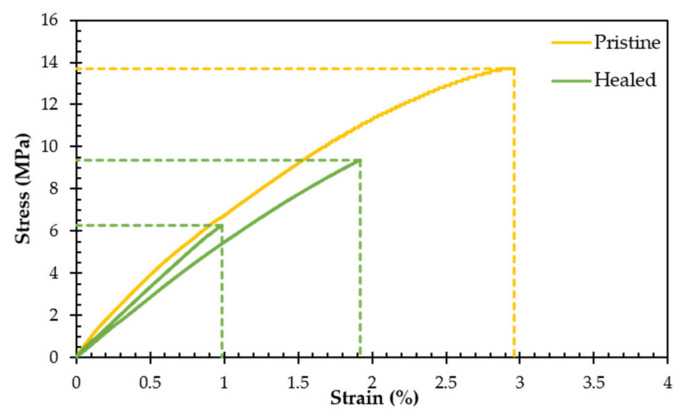
Stress–strain curve of DPBM-F400(*r* = 0.8) pristine and samples were cut completely and healed for 1 h at 90 °C.

Yet, the stiffer reversible polymer networks with higher stoichiometric ratios (*r* > 0.5) can still be healed at higher temperatures, as shown in Figure 10. Heating up the materials, above their glass transitions, leads to an increase in mobility as well as in kinetics (Equation (1)) making it possible to heal in a reasonable time frame, in the order of hours. This is illustrated by healing tests on DPBM-F400 (*r* = 0.8) tensile samples with gauge length of 10 mm, width of 5 mm and thickness of 2 mm. These samples were first broken in half as pristine samples in a tensile test with strain ramp of 1%s^−1^, which led to a brittle fracture at stresses around 14 MPa (Figure 10). Thereafter, the samples were brought back in contact and were submitted to a healing procedure that involved heating the samples to 90 °C for 1 h. After this healing procedure, tensile testing shows that the fracture stress is recovered with 57% and the fracture strain with 49%.

## 4. Discussion

A series of reversible covalent polymer networks was prepared based on a four-functional furan compound (F400) and a bismaleimide (DPBM) mixed in different maleimide-to-furan stoichiometric ratios. The relation between the stoichiometric ratio and the glass transition, the equilibrium gelation temperature and conversion, the mechanical properties at ambient temperature and the thermomechanical properties from the glassy to the rubbery state were experimentally obtained. Lowering the stoichiometric ratio led to a decrease in the amount of Diels–Alder adducts (reversible crosslinks) that could be formed at ambient temperature, resulting in a more flexible polymer networks with a lower glass transition temperature and Young’s modulus, an increase in the strain at break and a decrease in the stress at break. Recently, such off-stoichiometric materials were employed by the authors to create two thermally reversible covalent elastomers with different mechanical properties that could be joined using the same reversible Diels–Alder bonding chemistry to create multi-material tendon-driven [11] and pneumatic robotic actuators [10]. In the design of these actuators, the difference in mechanical properties was exploited to achieve the desired actuation behavior. Furthermore, it was shown how the interface between the two RPN was at least as strong as the weakest of the two materials and that applied damage and interfacial failure could be healed successfully. By changing the stoichiometric ratio of the DPBM-F400 system, a wide register of thermomechanical properties can be obtained, ranging from brittle, glassy thermosets to flexible, ductile elastomers at the room temperature. This allows to optimize the mechanical properties of the network to fit specific demands of various applications.

Changing the stoichiometric ratio between maleimide and furan further impacts the reaction equilibrium and the (de)gelation behavior. Lowering the stoichiometric ratio results in a shift of the reaction equilibrium towards higher conversions of the maleimide minority component. In parallel, the critical gel conversion increases with decreasing stoichiometric ratio. As the latter change is more pronounced than the shift in the equilibrium conversion, this finally results in lower gel transition temperatures at lower stoichiometric ratios. When comparing simulated and experimental gel transition temperatures, it was shown that at higher stoichiometric ratios the model is able to calculate the gel transition temperature very well, while the error becomes bigger as the stoichiometric ratio decreases, although it is still limited to 4 K at *r* = 0.4. Nevertheless, the simulation is an excellent tool to estimate the gel transition, proven to be useful in the determination of the temperature-time profile required for manufacturing, as illustrated by the authors [9] in a fused filament fabrication technique for an RPN based on the Diels–Alder reaction.

According to isothermal simulations for the bond formation process at 25 °C, the conversion rate is higher for the lowest stoichiometric ratios, approaching the equilibrium conversion considerably faster than near-stoichiometric reactive systems. This can be explained by the large excess of furan, speeding up the reaction of the maleimide that is in deficit. In contrast, the time to reach gelation increased for lower stoichiometric ratios, due to the higher critical gel conversion. Because of this higher conversion rate, healing is favoured as well in elastomers with low stoichiometry ratio, as illustrated by the healing at 25 °C, resulting in excellent recovery of the mechanical properties. Recently, the authors published the creation and healing evaluation of a pneumatically actuated soft robotic finger that possessed the ability to self-heal many types of damage under ambient conditions [12]. The ability of this DPBM-F5000 (*r* = 0.5) to heal under ambient conditions, as opposed to the stoichiometric (*r* = 1) system, can be explained based on the findings of the systematic study presented here. The lower concentration of adduct bonds (reversible crosslink density) provides the higher flexibility and mobility of the polymeric chains needed to achieve efficient healing, while the increased conversion rate due to the high excess of furan groups results in fast healing kinetics to reform the broken bonds at the damage surfaces. Thermosets, with higher stoichiometry, lack mobility and fast conversion rate, making it impossible to heal at ambient conditions. Nonetheless, heating damaged samples, leads to an increase in both mobility and kinetics, resulting in the ability to heal large macroscopic damages.

## 5. Conclusions

The effect of the maleimide-to-furan stoichiometric ratio on the crosslink density, glass transition temperature, mechanical properties, thermomechanical behavior (viscoelastic properties, gel transition temperature), Diels–Alder kinetics, and the self-healing behavior of a series of furan-maleimide thermoreversible networks was studied comprehensively by means of thermal analysis instruments (DSC, dynamic rheometry, and DMA) as well as using kinetics simulation. Lowering the stoichiometric ratio led to a decrease in the amount of reversible crosslinks, resulting in more flexible polymer networks with a lower glass transition temperature and Young’s modulus, an increase in the strain at break, and a decrease in the stress at break. According to kinetics simulation, by lowering the stoichiometric ratio, the gelation time decreases, while the reaction equilibrium and the critical gel conversion shift to higher conversions (at the same temperature). As a result, the decreasing stoichiometric ratio results in lower gel transition temperatures. Our work illustrated that starting from only two specific monomers, a bismaleimide (DPBM) and a furan functionalized Jeffamines (F400), a wide variety of polymer networks can be synthesized with mechanical properties ranging from very stiff thermoset to a hyperelastic elastomer that can heal at room temperature.

## Figures and Tables

**Table 1:** Initial concentration of maleimide and furan, equilibrium concentrations of maleimide, furan and Diels–Alder cycloadducts at 25 °C, and equilibrium conversions at 25 and 100 °C of synthesized DA polymeric networks with different maleimide to furan ratios (*r*).

Network	Initial Condition		Equilibrium Condition				
	[*M*]_0_ (mol kg^−1^)	[*F*]_0_ (mol kg^−1^)	[*DA*]*_eq_* (mol kg^−1^)	[*M*]*_eq_* (mol kg^−1^)	[*F*]*_eq_* (mol kg^−1^)	[*x*]*_eq_* at 25 °C	[*x*]*_eq_* at 100 °C
DPBM-F400 (*r* = 1.0)	2.231	2.231	2.149	0.082	0.082	0.963	0.710
DPBM-F400 (*r* = 0.8)	1.940	2.425	1.928	0.012	0.497	0.994	0.777
DPBM-F400 (*r* = 0.7)	1.775	2.535	1.768	0.007	0.768	0.996	0.807
DPBM-F400 (*r* = 0.6)	1.594	2.656	1.589	0.005	1.067	0.997	0.834
DPBM-F400 (*r* = 0.5)	1.394	2.789	1.391	0.003	1.397	0.998	0.858
DPBM-F400 (*r* = 0.4)	1.174	2.935	1.172	0.002	1.763	0.999	0.878

**Table 2:** Equilibrium concentration of Diels–Alder cycloadducts at 25 °C, glass transition temperature, Young’s modulus, fracture stress, and fracture strain of synthesized RPNs that differ in stoichiometric ratio *r*.

Network	[*DA*]*_eq_* at 25 °C (mol kg^−1^)	*T_g_* (°C)	Young’s Modulus (MPa)	Fracture Stress (MPa)	Fracture Strain (%)
DPBM-F400 (*r* = 1.0)	2.149	68	1755 ± 78	20.9 ± 1.8	1.58 ± 0.07
DPBM-F400 (*r* = 0.8)	1.928	51	844 ± 12	13.4 ± 0.2	2.0 ± 0.1
DPBM-F400 (*r* = 0.7)	1.768	46	584 ± 49	7.6 ± 0.3	2.1 ± 0.2
DPBM-F400 (*r* = 0.6)	1.589	38	216 ± 28	4.5 ± 1.6	3.4 ± 0.2
DPBM-F400 (*r* = 0.5)	1.391	16	9.8 ± 2.1	0.48 ± 0.06	156 ± 17
DPBM-F400 (*r* = 0.4)	1.172	2	0.23 ± 0.04	0.13 ± 0.01	317 ± 14

**Table 3:** Equilibrium crosslink density, storage modulus (*E’*), loss modulus (*E”*) and loss angle (*δ*) at 25 °C for the six RPNs that differ in stoichiometric ratio (*r*).

Network	[*DA*]*_eq_* (mol kg^−1^)	*E*′ (MPa)	*E*″ (MPa)	*δ*
DPBM-F400 (*r* = 1.0)	2.149	2023	208.2	4.68
DPBM-F400 (*r* = 0.8)	1.928	1974	161.7	5.87
DPBM-F400 (*r* = 0.7)	1.768	1896	218.7	6.58
DPBM-F400 (*r* = 0.6)	1.589	594.5	277.6	25.03
DPBM-F400 (*r* = 0.5)	1.391	208.8	119.5	29.79
DPBM-F400 (*r* = 0.4)	1.172	45.95	47.2	45.74

**Table 4:** Critical gel conversion, simulated equilibrium and non-equilibrium gel temperature, experimentally derived gelation temperature, simulation of gelation time and required time to reach 95% conversion at 25 °C for RPN with different stoichiometric ratios *r*.

Network	*x_gel_*	*T*_gel,sim (eq)_ (°C)	*T*_gel,sim (non-eq)_ (°C)	*T*_gel,exp_ (°C)	*t*_gel, 25 °C_ (h)	*t*_95% eq, 25 °C_ (h)
DPBM-F400 (*r* = 1.0)	0.577	121.7	122.5	124.0	2.83	216
DPBM-F400 (*r* = 0.8)	0.645	120.0	120.9	123.0	3.00	18.3
DPBM-F400 (*r* = 0.7)	0.690	117.9	118.9	121.5	3.17	12.5
DPBM-F400 (*r* = 0.6)	0.745	114.2	115.4	120.5	3.33	10.0
DPBM-F400 (*r* = 0.5)	0.816	107.4	109.5	113.0	4.00	8.33
DPBM-F400 (*r* = 0.4)	0.913	91.8	97.6	102.0	5.28	6.94

## Appendix A

**Table A1:** Kinetic parameters for the Diels–Alder reaction between DPBM and FXXX.

Kinetics Parameter	Units	Endo Isomer	Exo Isomer
ln(A_DA,i_)	kg.mol^−1^ s^−1^	14.3	15
E_DA,i_	kj mol^−1^	60.3	65.3
ln(A_rDA,i_)	s^−1^	30.2	31.8
E_rDA,i_	kJ mol^−1^	108	121
Δ_r_H^0^_i_	kJ mol^−1^	−47.7	−55.7
Δ_r_S^0^_i_	J mol^−1^ K^−1^	−133	−140
